# Effect of kilovoltage and quality reference mAs on CT-based attenuation correction in ^177^Lu SPECT/CT imaging: a phantom study

**DOI:** 10.1186/s40658-024-00622-6

**Published:** 2024-02-26

**Authors:** Maikol Salas-Ramirez, Julian Leube, Michael Lassmann, Johannes Tran-Gia

**Affiliations:** Department of Nuclear Medicine, University Hospital Würzburg, Oberdürrbacher Str. 6, 97080 Würzburg, Germany

**Keywords:** Quantitative SPECT/CT, Attenuation correction, CTDI_vol_, ^177^Lu SPECT/CT

## Abstract

**Introduction:**

CT-based attenuation correction (CT-AC) plays a major role in accurate activity quantification by SPECT/CT imaging. However, the effect of kilovoltage peak (kVp) and quality-reference mAs (QRM) on the attenuation coefficient image (*μ*-map) and volume CT dose index (CTDI_vol_) have not yet been systematically evaluated. Therefore, the aim of this study was to fill this gap and investigate the influence of kVp and QRM on CT-AC in ^177^Lu SPECT/CT imaging.

**Methods:**

Seventy low-dose CT acquisitions of an Electron Density Phantom (seventeen inserts of nine tissue-equivalent materials) were acquired using various kVp and QRM combinations on a Siemens Symbia Intevo Bold SPECT/CT system. Using manufacturer reconstruction software, ^177^Lu *μ*-maps were generated for each CT image, and three low-dose CT related aspects were examined. First, the *μ*-map-based attenuation values (*μ*_measured_) were compared with theoretical values (*μ*_theoretical_). Second, changes in ^177^Lu activity expected due to changes in the *μ*-map were calculated using a modified Chang method. Third, the noise in the *μ*-map was assessed by measuring the coefficient of variation in a volume of interest in the homogeneous section of the Electron Density Phantom. Lastly, two phantoms were designed to simulate attenuation in four tissue-equivalent materials for two different source geometries (1-mL and 10-mL syringes). ^177^Lu SPECT/CT imaging was performed using three different reconstruction algorithms (xSPECT Quant, Flash3D, STIR), and the SPECT-based activities were compared against the nominal activities in the sources.

**Results:**

The largest relative errors between *μ*_measured_ and *μ*_theoretical_ were observed in the lung inhale insert (range: 18%-36%), while it remained below 6% for all other inserts. The resulting changes in ^177^Lu activity quantification were -3.5% in the lung inhale insert and less than -2.3% in all other inserts. Coefficient of variation and CTDI_vol_ ranged from 0.3% and 3.6 mGy (130 kVp, 35 mAs) to 0.4% and 0.9 mGy (80 kVp, 20 mAs), respectively. The SPECT-based activity quantification using xSPECT Quant reconstructions outperformed all other reconstruction algorithms.

**Conclusion:**

This study shows that kVp and QRM values in low-dose CT imaging have a minimum effect on quantitative ^177^Lu SPECT/CT imaging, while the selection of low values of kVp and QRM reduce the CTDI_vol_.

**Supplementary Information:**

The online version contains supplementary material available at 10.1186/s40658-024-00622-6.

## Introduction

One of the essential steps in nuclear medicine hybrid imaging (PET/CT or SPECT/CT) is the attenuation correction (AC) of the acquired SPECT or PET data using X-ray CT scans. CT scans represent the attenuation coefficients (*μ*) of tissues or materials expressed in Hounsfield units (HUs), which are obtained by X-ray transmission measurements in the axial plane of a patient or object [1, 2]. To perform AC on SPECT or PET images, the CT image (in HUs) is converted to a map of attenuation coefficients (*μ*-map) for the gamma emission of the radionuclide used for imaging [3]. The most general approach to perform this conversion is based on a bilinear model relating HUs to the attenuation coefficients [1, 3]. However, each manufacturer has implemented different corrections to improve the accuracy of the *μ*-map for different clinical situations (e.g., corrections for image acquisition using multiple energy windows, scatter compensation due to the chosen energy window width, or in the presence of contrast agents) [4, 5].

CT imaging in PET/CT or SPECT/CT comes at the cost of increased radiation exposure for the patient [6]. Therefore, optimizing the CT acquisition protocol to reduce patient exposure while maintaining the required image quality and without affecting activity quantification is essential. In addition, large variations in the diagnostic reference levels (DRLs) for CT scans in hybrid imaging have been observed, raising the need for harmonizing the CT acquisition protocol in SPECT/CT and PET/CT [7–9].

Furthermore, the growing number of newly introduced radiopharmaceuticals has led to an increase in the use of quantitative ^177^Lu SPECT/CT. This increased utilization is primarily for the generation of time-activity curves for internal dosimetry [10–12]. Therefore, the establishment of evaluation methods to optimize the CT image acquisition protocol used for CT-AC in ^177^Lu SPECT/CT imaging is essential. In this context, the influence of the x-ray tube potential (V, typically in units of kV) and the product of tube current (*J*, typically in units of mA) and tube on-time per rotation (τ, typically in units of s) (*J*τ, mAs) [2] on CT-AC in ^177^Lu SPECT/CT imaging is a topic of current interest and complementary to state-of-the-art quantitative SPECT/CT guidelines [13, 14].

The objective of this study is to evaluate the influence of kilovoltage peak (kVp) and quality reference mAs (QRM) on attenuation coefficient map (*μ*-map), volume CT dose index (CTDI_vol_), and quantitative accuracy in quantitative ^177^Lu SPECT/CT imaging. The study is divided into four sections: The first two sections focus on the CT part by evaluating the accuracy of the methodology for converting HUs into µ-maps implemented in the Siemens Intevo Bold SPECT/CT system. The evaluation was based on an Electron Density Phantom composed of sections with various tissue-equivalent materials to assess changes in the image-based attenuation coefficient. In the third section, image noise as a selection criterion for optimizing CT-absorbed doses is evaluated by assessing the image noise in the water-equivalent homogeneous section of the Electron Density Phantom. In the fourth and last section, the impact of CT parameters on ^177^Lu SPECT/CT image quantification is analyzed for different reconstruction algorithms. Moreover, quantitative ^177^Lu SPECT/CT imaging was performed for two different source geometries using different materials to mimic different clinically realistic attenuation conditions.

Although the radiation exposure from CT during therapeutic planning procedures for ^177^Lu-PSMA or ^177^Lu-DOTATOC therapy is typically minimal compared to the therapeutic absorbed dose, this study places a strong emphasis on adhering to the radiation protection principle of optimization and achieving accurate quantification of ^177^Lu in internal dosimetry procedures [7, 15]. These efforts are critical for compliance with national diagnostic reference levels (DRLs) in the context of hybrid imaging, but also for optimization of total CT doses in internal dosimetry procedures, which should ideally be performed based on multiple SPECT/CT time points. Importantly, the methodology developed in the study may not be limited to ^177^Lu SPECT/CT, but has broader applicability to other radionuclides used in hybrid imaging.

## Materials and methods

CT images of the CT Electron Density Phantom Model 062 M (computerized imaging reference systems, CIRS) were acquired with a SPECT/CT system (Symbia Intevo Bold, Siemens Healthineers) with adjustable CT voltage (80 kVp, 110 kVp, and 130 kVp). All CT reconstructions were performed with the I31s kernel (Sinogram AFfirmed Iterative REconstruction, SAFIRE at strength S3), which utilizes a weighted FBP for initial reconstruction, followed by two correction loops [16]. The Segment Editor module of 3D Slicer (version 4.8.1) [18, 19] was used to analyze the CT images. All mathematical calculations were performed in Python (version 3).

### Comparison between CT-based linear attenuation coefficients and the national institute of standards and technology linear attenuation coefficients database

**Fig. 1:**
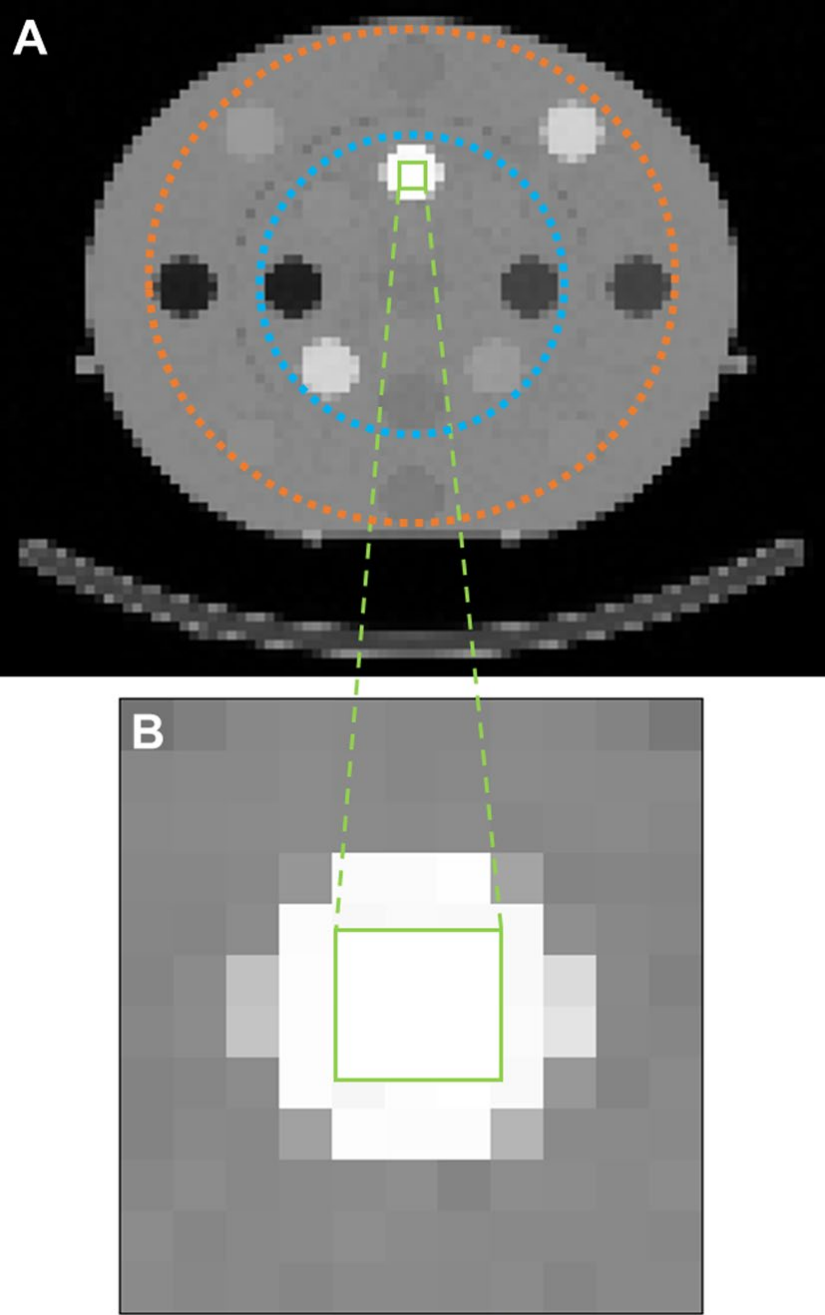
Positioning of the cubic volume of interest of 3 × 3 × 8 voxels in the bone insert with 1250 mg/cm^3^ hydroxyapatite in 130 kVp and 35 mAs image. **A** whole phantom. Blue dotted line indicates position of inner inserts, orange dotted line indicates position of outer inserts. **B** Zoom over the bone insert and placing of the cubic volume of interest

Seventy CT acquisitions of the Electron Density Phantom equipped with seventeen inserts of nine different tissue-equivalent materials (exhale lung, inhale lung, adipose, breast, muscle, liver, bone 200 mg/cm^3^ hydroxyapatite (HA), bone 800 mg/cm^3^ HA, and bone 1250 mg/cm^3^ HA) were acquired. These measurements included seven different combinations of kVp and QRM (80kVp with 20mAs, 35mAs, and 50mAs, 110kVp with 20mAs and 35mAs, and 130kVp with 20mAs, and 35mAs), each repeated ten times. All acquisitions were performed with a pitch of 1.5. A ^177^Lu *μ*-map based on the I31s CT reconstruction kernel was generated using the standard manufacturer reconstruction (Flash3D, Siemens Healthineers) with a voxel size of 4.8 mm. The mean *μ* value (*μ*_measured_) of each insert was quantified in cubic volumes of interest (VOIs) of 3 × 3 × 8 voxels (Fig. 1). To identify a potential radial dependency of the attenuation (e.g., due to the CT beam hardening conditions), the *μ*_measured_ values for the inserts located on the inner and outer section of the Electron Density Phantom were evaluated independently: two datasets (dataset 1: nine inner inserts excluding the 1250 mg/cm^3^ HA bone insert; dataset 2: eight outer inserts) were used for statistical tests of normality (Shapiro–Wilk normality test) and mean difference (unpaired t-test). Next, for each insert, *μ*_measured_ was compared against the theoretical *μ* value (*μ*_theoretical_) for 208.4 keV ^177^Lu gamma emission (peak with highest emission probability) from the National Institute of Standards and Technology (NIST) standard reference database 8 [17], which allows the calculation of the attenuation coefficient of a mixture (a combination of elements) using the elemental material composition and mass density provided by the manufacturer of the Electron Density Phantom [18]. Moreover, the CTDI_vol_ for each combination of kVp and QRM was extracted from the DICOM header of each image.

### Calculation of the attenuation correction factor image for ^177^Lu from the *μ*-map

**Fig. 2:**
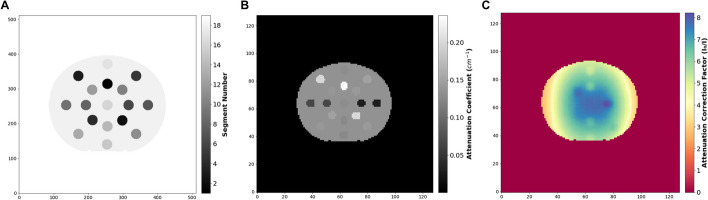
Digital phantom from the CIRS electron density phantom. **A** Segments from the high resolution CT image. **B** µ-map based on theoretical values from NIST database [17]. **C** Theoretical attenuation correction factor (ACF_theoretical_) image. $I_{0}$ is the image with attenuation correction and $I$ is the image without attenuation correction

In this section, a theoretical approach was applied to evaluate the influence of the kVp and QRM on ^177^Lu SPECT/CT-based activity quantification. Specifically, the Chang algorithm [19] was implemented using measured ^177^Lu *μ*-maps in combination with the equation described in Bailey et al*.* [20]. First, to create a theoretical *μ*-map, a digital version of the Electron Density Phantom was generated based on one of the 130 kVp and 35 mAs CT images of 512 × 512 × 86 voxels with a voxel size of 0.977 × 0. 977 × 3 mm^3^. All inserts, the contour of the phantom, and all voxels outside the phantom (air plus bed) were separately segmented in 3D Slicer (Fig. 2A). Then, these segmentations were downscaled to the voxel size (4.8 mm) of the *μ*-map (128 × 128 × 55). Next, the *μ*_theoretical_ values for the 208.4 keV ^177^Lu gamma emission were assigned to each segment (19 segments comprising 17 material inserts, the water plastic phantom material, and air) using the 3D Slicer segmentation color table (Fig. 2B).

Second, to generate the attenuation correction factor (ACF) images, an existing Python script [21] was extended to implement a modified Chang method [20] using the theoretical *μ*-map (ACF_theoretical_) (Fig. 2C and Additional file 2: material) and measured *μ*-maps for each kVp and QRM combination (generated in section 1 of “Materials and methods”) (Additional file 3: material).

Next, ACF_measured_ and ACF_theoretical_ were analyzed using the same VOI sizes and localizations as defined in section 1 of “Materials and methods”. Subsequently, the change in ^177^Lu activity quantification ($\Delta A\left[ \% \right]$) to be expected was assessed using Eq. 1 (derivation in Additional file 1):

1 $$\Delta A\left( {{}_{{}}^{{{177}}} {\text{Lu}}} \right){\text{[\% ] = }}\left( {\frac{{{\text{ACF}}_{{{\text{theoretical}}}} }}{{{\text{ACF}}_{{{\text{measured}}}} }}{ - 1}} \right) \cdot {\text{100\% }}$$

Lastly, a Shapiro–Wilk normality test followed by a one-way analysis of variance (parametric or nonparametric depending on the normality test) for comparing multiple independent samples were performed to compare the changes in ^177^Lu activity quantification based on the seven kVp and QRM combinations.

### Noise assessment in CT-based ^177^Lu *μ*-map

**Fig. 3:**
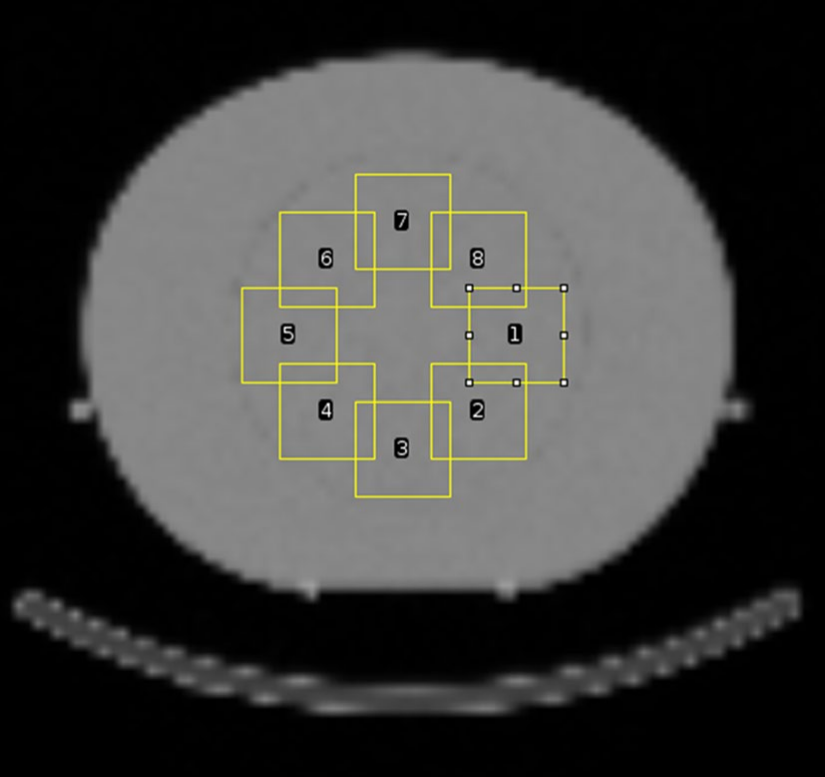
Segmented VOIs for the noise analysis. Illustration performed on imageJ [28]

To evaluate the influence of kVp and QRM of the CT-AC on the noise in the attenuation map, eight cubic VOIs of 1,000 voxels each were drawn on the homogeneous plastic water section of the Electron Density Phantom in the CT-AC-based ^177^Lu *μ*-map (the positioning is described in Fig. 3). This process was repeated for all ten repetitions of each kVp and QRM combination. First, a mean *μ* value $\left( {\mu_{{{\text{mean}}}} } \right)_{j}$ over all eight cubic *i* VOIs was calculated for each repetition *j*:

2 $$\left( {\mu_{{{\text{mean}}}} } \right)_{j} = \frac{{\mathop \sum \nolimits_{i = 1}^{8} \left( {\mu_{{{\text{VOI}}}} } \right)_{i,j} }}{8}$$

Subsequently, total mean ($\mu_{{{\text{mean}}}}$) and standard deviation ($\mu_{{{\text{SD}}}}$) over all ten repetitions *j* was calculated for each combination of kVp and QRM:

3 $$\mu_{{{\text{mean}}}} = \frac{{\mathop \sum \nolimits_{j = 1}^{10} \left( {\mu_{{{\text{mean}}}} } \right)_{j} }}{10},$$

4 $$\mu_{{{\text{SD}}}} = \sqrt {\frac{1}{9}\mathop \sum \limits_{j = 1}^{10} \left( {\left( {\mu_{{{\text{mean}}}} } \right)_{j} - \mu_{{{\text{mean}}}} } \right)^{2} }$$

Finally, the coefficient of variation (COV) was calculated as:

5 $${\text{COV}} = \frac{{\mu_{{{\text{SD}}}} }}{{\mu_{{{\text{mean}}}} }}$$

### Influence of different attenuation materials on activity quantification accuracy

**Fig. 4:**
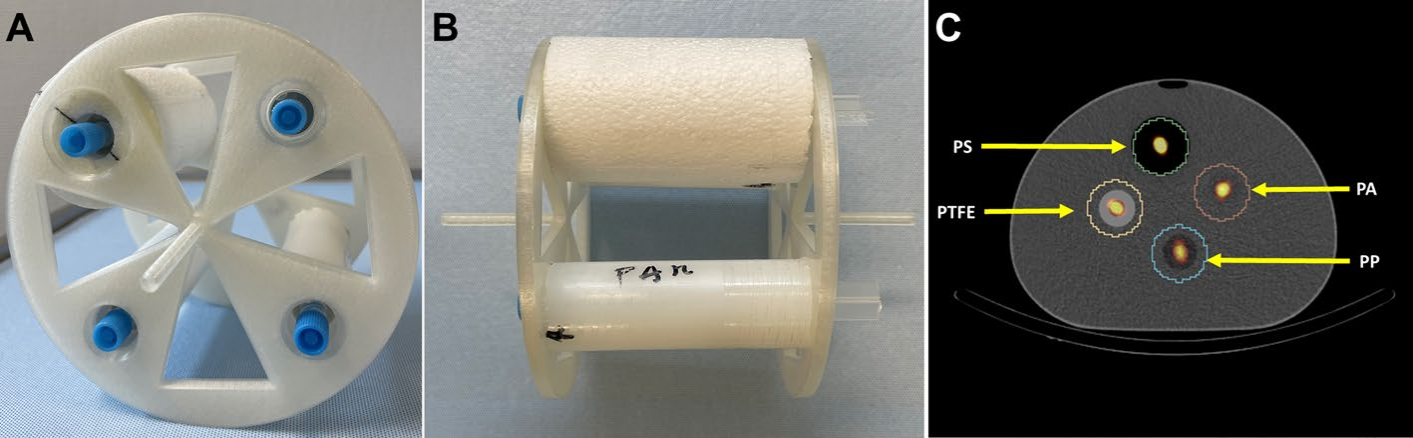
Quantitative SPECT: **A** Frontal view of 10-mL syringe insert. **B** Lateral view of 10-mL syringe insert. **C** Segmentation boundaries using 10-mL syringe insert with sources inside of NEMA Phantom

In this section, the influence of kVp, QRM, and the SPECT reconstruction algorithm on the SPECT/CT-based ^177^Lu quantification was evaluated experimentally. To compare different attenuation materials, a phantom consisting of four radioactive sources (syringes of volume 1 mL / diameter 0.7 cm or 10 mL / diameter 1.6 cm), each surrounded by a different attenuation medium, was designed. It consisted of the IEC NEMA body phantom container and a 3D-printed mounting system for four cylinders made of different attenuation materials, each of which had a hollow cylinder drilled in its center for axial syringe insertion (Fig. 4A and B). The attenuation materials included lung (Polystyrene, PS, diameter: 5 cm, density: 0.023 g/cm^3^), bone (polytetrafluoroethylene, PTFE, diameter: 3 cm, density: 2.18 g/cm^3^), soft tissue (polyamide, PA, diameter: 3 cm, density: 1.02 g/cm^3^), and fat tissue equivalent materials (polypropylene, PP, diameter: 3 cm, density: 0.91 g/cm^3^). These densities are based on the manufacturer specifications [22, 23]. To compensate for the low mass density of Polystyrene, a cylinder with a larger diameter was used in comparison to the other three cylinders.

A 100-mL radioactive stock solution consisting of ^177^Lu-chloride dissolved in 0.1 M HCl with 100 ppm of stable lutetium [13] was prepared. Eight syringes (four syringes of 1 mL and four syringes of 10 mL) were weighted before and after the filling process for a weight-based determination of the contained activity. A VDC-405 radionuclide calibrator equipped with a VIK-202 ionization chamber (Comecer SpA) was used for estimating the activity added to the stock solution during the phantom filling process. The reference activity concentration was obtained from a 1-mL aliquot of the stock solution, which was measured in a high-purity germanium (HPGe) detector (model GR4020 [Canberra]), whose energy-dependent efficiency had previously been calibrated with several NIST-traceable standards over the energy range considered [13].

SPECT/CT images of both phantom setups (four 1-mL syringes, four 10-mL syringes) placed inside a water-filled (to simulate soft tissue attenuation) IEC NEMA body phantom were acquired (Fig. 4C). The images were acquired with a Siemens Intevo Bold SPECT/CT system with 9.5-mm crystal thickness, medium-energy low-penetration collimation, 180° detector configuration, automatic contouring, continuous mode, 60 views, 30 s per view, 256 × 256 matrix, and 3 energy windows (20% around the main photopeak of 208 keV and two adjacent 10% scatter windows). After SPECT acquisition, seven CT acquisitions were performed using the same combinations of kVp and QRM as described in the previous Sects. (80kVp with 20mAs, 35mAs, and 50mAs, 110kVp with 20mAs and 35mAs, and 130kVp with 20mAs, and 35mAs). The CT-AC images were reconstructed with the I31s kernel in a 512 × 512 × 133 matrix with a resolution of 1.0 × 1.0 × 3.0 mm^3^. Next, 7 × 3 SPECT reconstructions were performed, one for each kVp and mAs combination (seven CT-based *μ*-maps) using three different reconstruction algorithms: (1) *xSPECT Quant* (Siemens Healthineers), an ordered-subset conjugate gradient minimization (OSCGM) reconstruction providing images in activity concentration (Bq/mL) based on a NIST traceable calibration source; the calibration is performed as part of the quality controls of the SPECT/CT system; reconstruction was performed using 24 iterations, 1 subset, and no postfilter [24]. (2) *Flash3D* (Siemens Healthineers), ordered-subset expectation maximization (OSEM) with attenuation correction, scatter correction, and depth-dependent 3D resolution recovery, which generates reconstructed images in counts; as recommended by the manufacturer, images were downsampled to a matrix size of 128 (voxel size: 4.8 mm); reconstruction was performed using 6 iterations, 6 subsets and no postfilter; an image calibration factor (ICF) of 20.3 cps/MBq (counts-per-second-per-Megabecquerel) as described in Tran-Gia et al*.* [13] for the same setup was used for conversion from counts to activity concentration. (3) STIR (Open Source software) [25], OSEM with attenuation correction, scatter correction, and a depth-dependent 3D resolution recovery method; as for Flash3D, images were downsampled to a matrix size of 128; reconstructions were performed using 6 iterations, 6 subsets and no postfilter; here, an ICF of 20.0 cps/MBq (determined as described by Tran-Gia et al*.* [13]) was used for conversion from counts to activity concentration.

After SPECT reconstruction, cylindrical VOIs were drawn on the 130 kVp and 35 mAs CT images of each phantom. In addition to the syringes and the attenuation material, the VOIs included were extended to a portion of the water phantom (Fig. 4C) to account for the activity distributed outside the walls of the syringe due to the limited spatial resolution of SPECT imaging [26]. These VOIs were applied to all SPECT reconstructions to ensure that the evaluation of activity was performed comparably between the different reconstructions.

## Results

### Comparison between CT-based linear attenuation coefficients and the national institute of standards and technology linear attenuation coefficients database

**Fig. 5:**
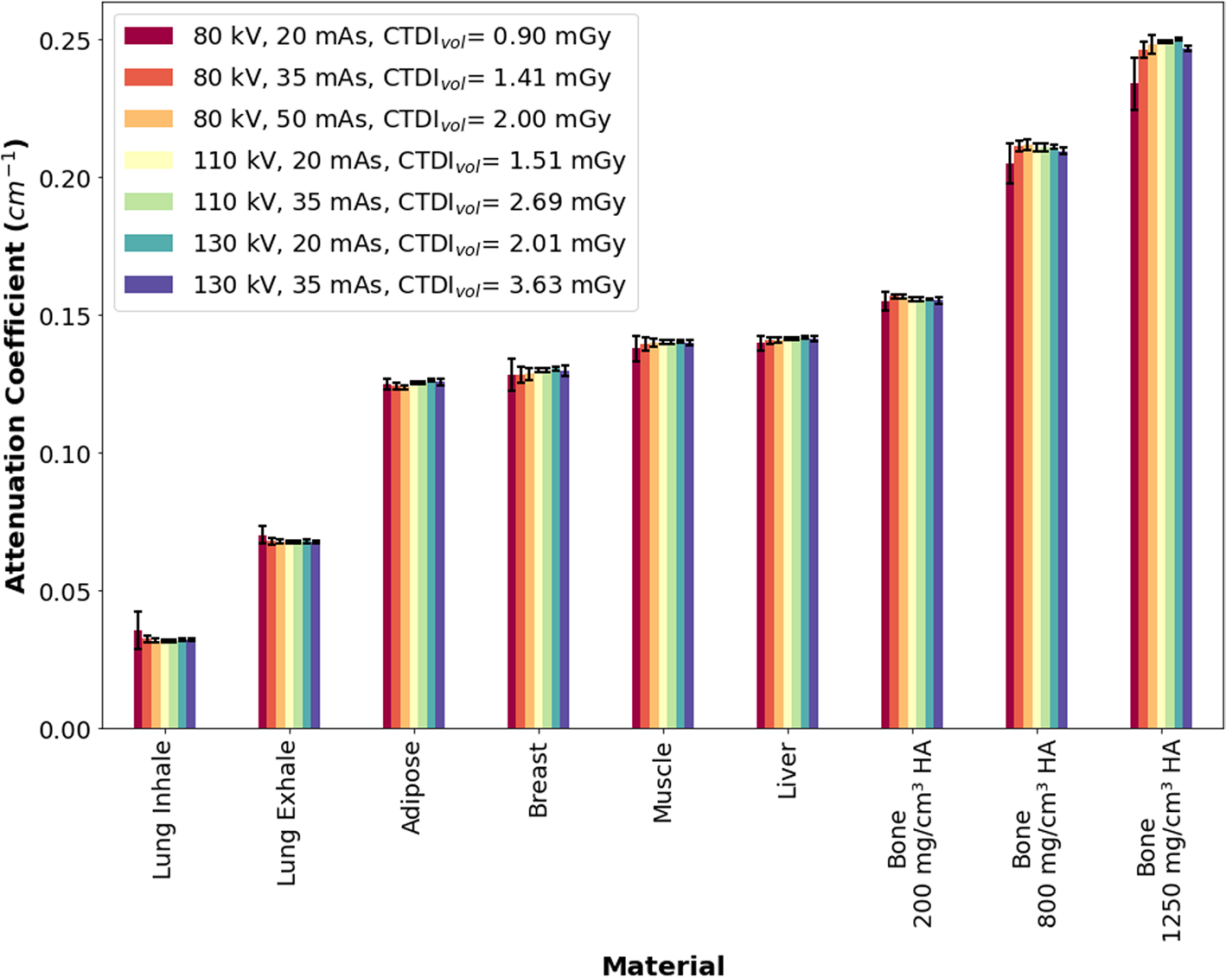
Mean measured attenuation coefficients (*µ*_measured_). Error bars consider a coverage factor (*k*) of 2

According to a Shapiro–Wilk normality test, all data (inner inserts excluding the 1,250 mg/cm^3^ HA bone insert and outer inserts) were normally distributed. Based on an unpaired t-test, no statistical difference (*p* > 0.05) was observed between *μ*_measured, inner_ and *μ*_measured, outer_ (inserts located in the inner and outer section of the Electron Density Phantom). Additional file 1: Fig. S1 shows data separated into inner and outer inserts, while Fig. 5 shows *μ*_measured, mean_ calculated as:

6 $$\mu_{{{\text{measured,}}\;{\text{mean}}}} = \left( {\mu_{{{\text{measured,}}\;{\text{inner}}}} + \mu_{{{\text{measured,}}\;{\text{outer}}}} } \right)/2$$

**Fig. 6:**
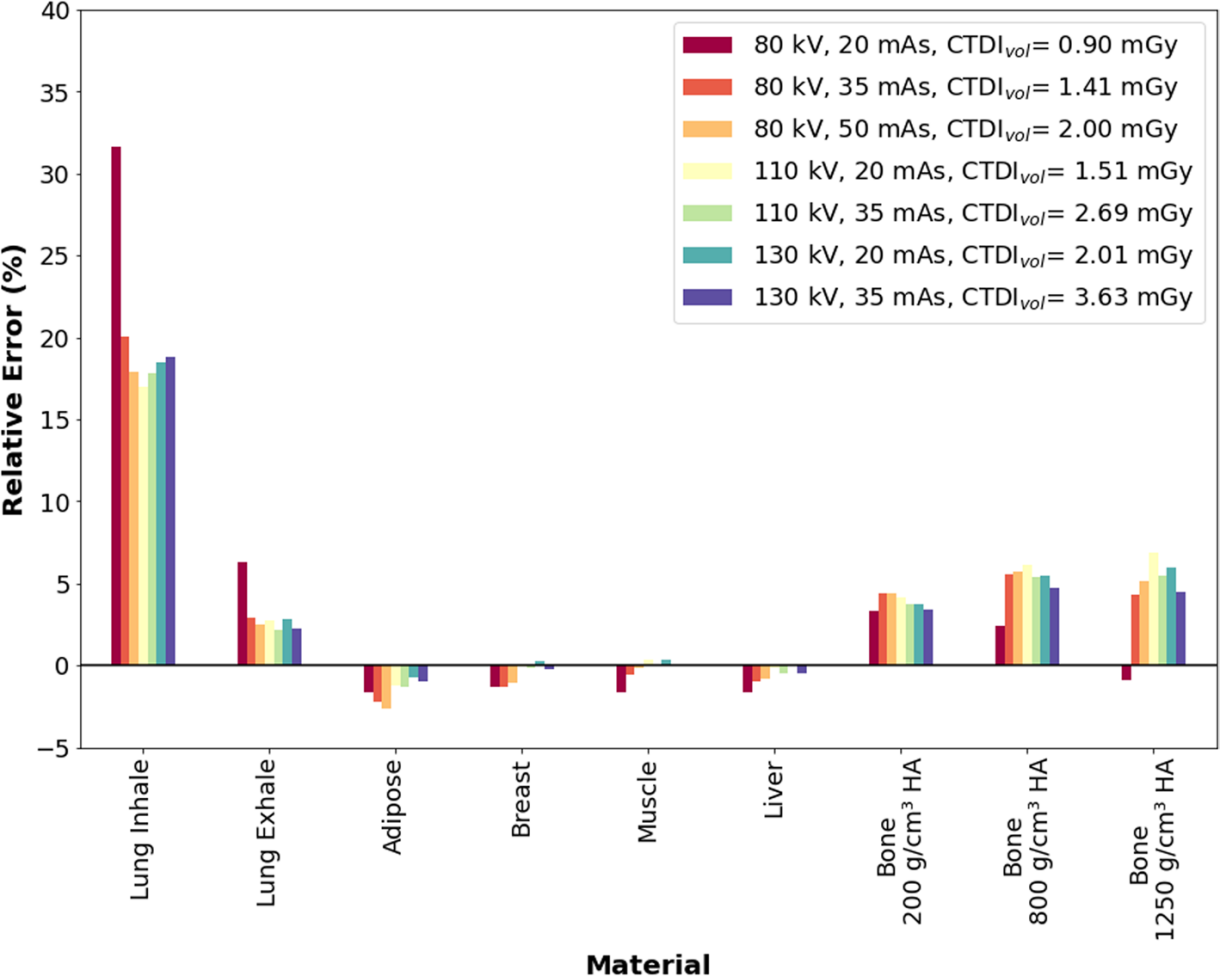
Relative error between the mean measured attenuation coefficients (*µ*_measured_) and the theoretical attenuation coefficients (*µ*_theoretical_). Error bars consider a coverage factor (*k*) of 2

Figure 6 shows the differences between *μ*_measured, mean_ and *μ*_theoretical_ (i.e., based on the NIST database). The largest difference of 32% between *μ*_measured, mean_ and *μ*_theoretical_ was observed in the lung inhale insert (80 kVp, 20 mAs). For lung exhale and bone, an overestimation by ~ 5% relative to *μ*_theoretical_ was observed. For all other soft tissues inserts (adipose, breast, muscle, and liver), the differences were less than 3%. Additional file 1: Figure S2 shows data separated into inner and outer inserts.

### Calculation of the attenuation correction factor image for ^177^Lu from the *μ*-map

The ACF values in the analyzed VOIs showed few variations for the seven tested kVp and QRM combinations. Additional file 1: Figure S3 illustrates the calculated values separated into inner and outer inserts. For the inner inserts, the ACF ranged between 6.8 (adipose tissue for 80 kVp, 20 mAs) and 8.3 (lung inhale tissue, 110 kVp, 20 mAs). These values are in agreement with the expected ACF of a voxel in a plastic water phantom (as for the Electron Density Phantom) placed in a depth of 13 cm (half of the anterior–posterior dimension of the electron density phantom) or a depth of 16 cm (half of the lateral dimension of the electron density phantom): ACF_13cm_ = 5.8 and ACF_16cm_ = 8.7, respectively. For the outer inserts, the ACF ranged between 4.9 (lung exhale, 110 kVp, 20 mAs) and 6.6 (liver, 80 kVp, 50 mAs), which are smaller than the values of the inner samples, as expected.

**Fig. 7:**
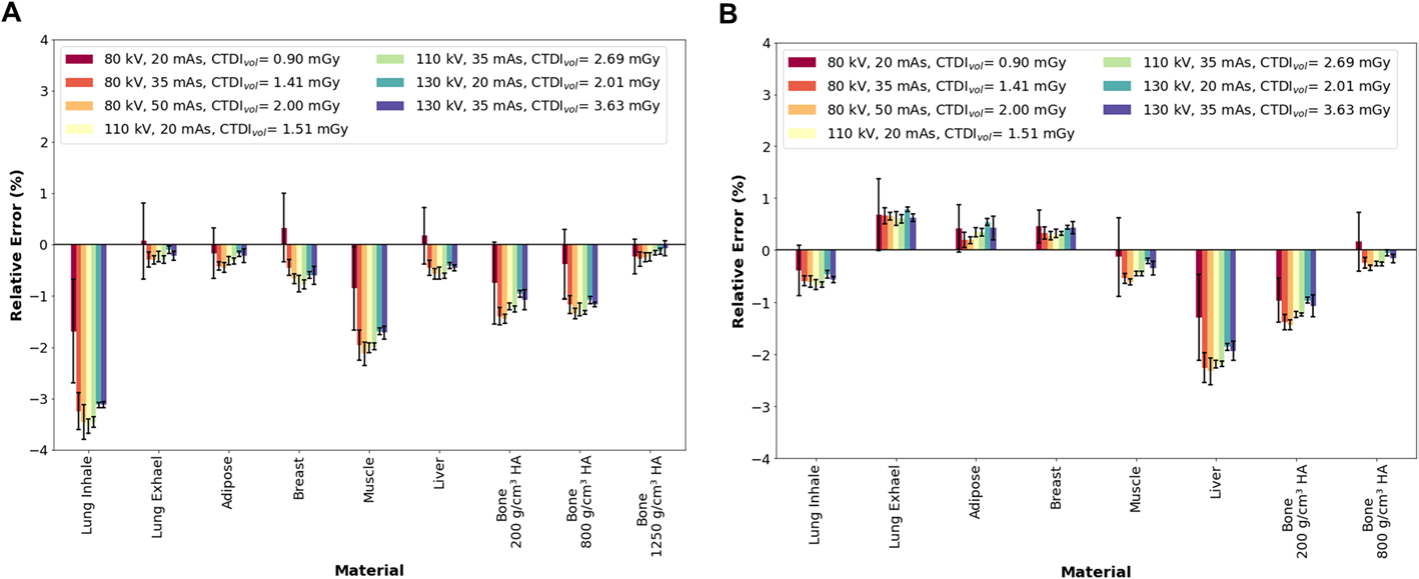
Relative error between the attenuation correction factor (ACF_measured_) and the theoretical attenuation correction factor (ACF_theoretical_) for: **A** Insert located in the inner section of the phantom. **B** Inserts located in the outer section of the phantom. Error bars consider a coverage factor (*k*) of 2

The maximum calculated change in ^177^Lu activity quantification using Eq. 1 for the inner inserts was obtained for lung inhale with − 3.5% (110 kVp, 20 mAs), followed by muscle, bone 200 g/m^3^ hydroxyapatite (HA), and bone 800 g/cm^3^ HA with − 2.1% (80 kVp, 50 mAs), − 1.5% (80kVp, 50mAs), and − 1.3 (80 kVp, 50 mAs), respectively. For the outer inserts, maximum differences were calculated for liver and bone 200 g/m^3^ HA with − 2.1% (80 kVp, 50 mAs) and − 1.5% (80 kVp, 50 mAs), respectively. Figure 7 shows the relative error for inner and outer inserts.

Kruskal–Wallis tests (for non-normally distributed data) revealed non-significant differences (*p* = 0.36 for inner inserts and *p* = 0.93 for outer inserts) between the different combinations of kVp and QRM.

### Noise assessment in CT-based ^177^Lu *μ*-map

**Fig. 8:**
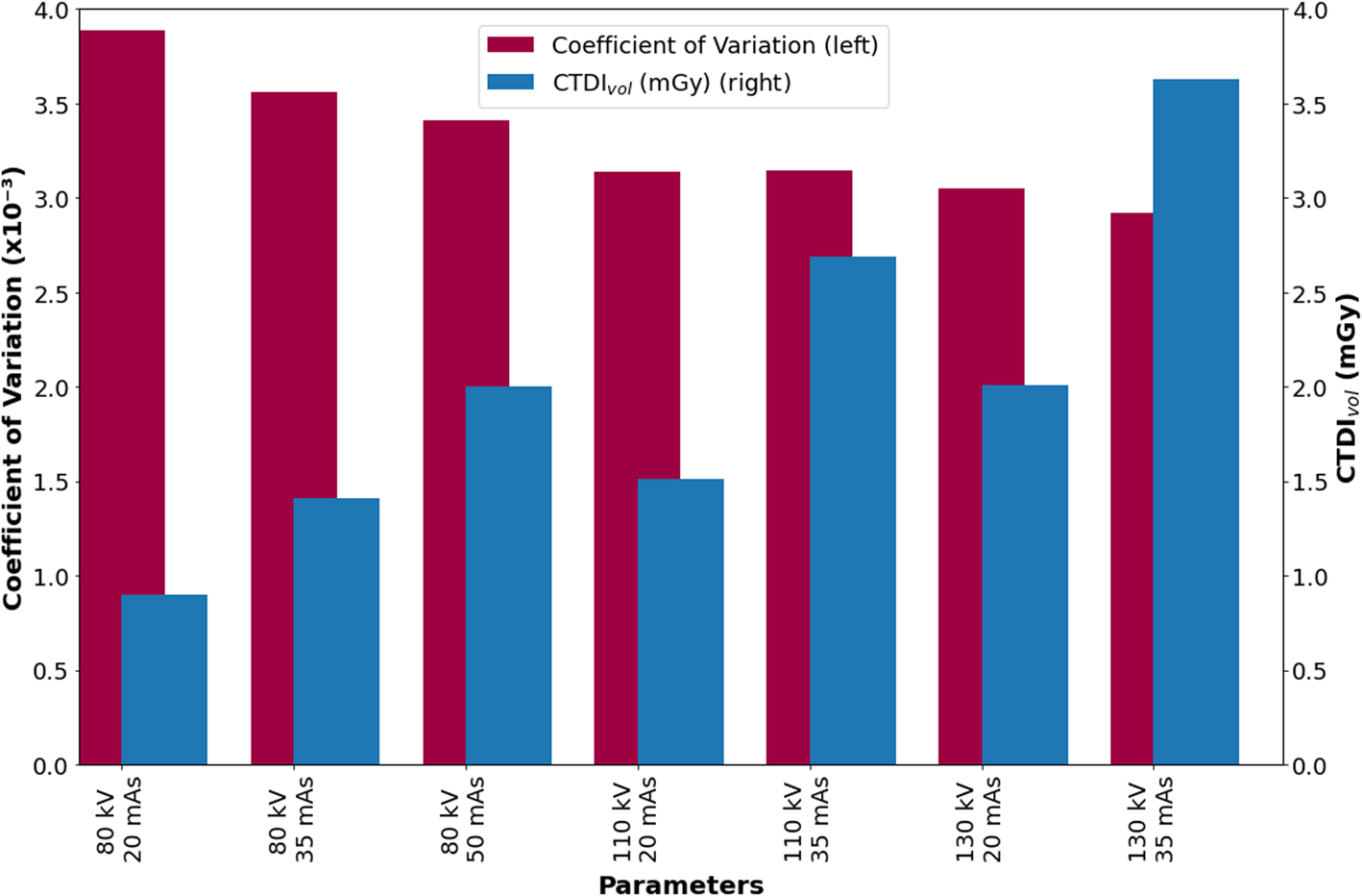
Noise analysis. Measured noise coefficient of variation together with the mean CTDI_vol_ for each kVp and QRM combination

The mean attenuation coefficient ranged between 0.1352 cm^−1^ (130 kVp, 20 mAs) and 0.1355 cm^−1^ (80 kVp, 50 mAs). These values are in very good agreement with the theoretical value of 0.1355 cm^−1^ for plastic water (PW-LR material [18], Electron Density Phantom) at 208.4 keV ^177^Lu gamma emission. The coefficient of variation and CTDI_vol_ ranged from 2.9 × 10^–3^ and 3.6 mGy (130 kVp, 35 mAs) to 3.9 × 10^–3^ and 0.9 mGy (80 kVp, 20 mAs), respectively (see Fig. 8).

### Quantitative SPECT—quantification of activity attenuated by four different materials

The errors of ^177^Lu SPECT-based activity quantification varied across the different reconstruction algorithms and inserts. Percentage errors of − 13%, 53%, and 60% represent the largest discrepancies for the 1-mL syringe in the PTFE insert (xSPECT Quant), the PS cylinder (STIR), and the PTFE insert (Flash3D), respectively. In contrast, the percentages of − 7%, 40%, and 40% correspond to the smallest errors observed for the PA insert (xSPECT Quant), the PP insert (Flash3D), and the PP insert (STIR). Increasing the source size from 1 to 10 mL improved the overall activity quantification based on ^177^Lu SPECT/CT. For xSPECT Quant, the maximum error was 9% (PTFE insert), for Flash3D it was 22% (PA insert), and for STIR it was 13% (PA insert). Furthermore, the activity quantification within the PTFE insert (Flash3D) and the PS insert (STIR) showed the most significant improvement.

**Fig. 9:**
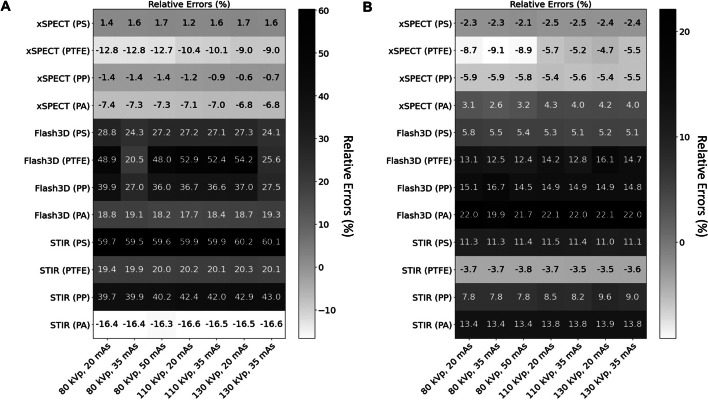
Relative error matrix between the measured and nominal activity for the four tested reconstruction algorithm and attenuation material of: **A** 1-mL syringe sources and **B** 10-mL syringe sources. Materials: *PS* Polystyrene, PTFE Polytetrafluoroethylene, *PA* Polyamide, and *PP* Polypropylene

For both source sizes, xSPECT Quant outperformed all other reconstructions with relative errors lower than -13%. In addition, xSPECT Quant showed less dependence on the syringe size than Flash3D and STIR. Furthermore, the relative errors for the 1-mL syringe inside the PTFE insert showed a similar change (4%) within the studied combinations of kVp and QRM (from ‑13% for 80 kVp / 35 mAs to -9% for 130 kVp / 35 mAs) as the 10-mL syringes (from -9% for 80 kVp / 35 mAs to − 5% for 130 kVp / 20 mAs). The relative error associated with the ^177^Lu SPECT-based activity quantification for 1-mL and 10-mL syringes is illustrated in Fig. 9 and Additional file 1: Figure S4. The nominal activities for each syringe are documented in Table S1 of the Additional file 1.

## Discussion

A good agreement was found between measured attenuation coefficients (*μ*_measured_) obtained from reconstructed ^177^Lu *μ*-maps to theoretical attenuation coefficients (*μ*_theoretical_) for all attenuation materials except lung inhale tissue. The same result was observed when evaluating the change in ^177^Lu activity (Eq. 1) using ACF_measured_ and ACF_theoretical_ with the largest error in activity quantification again observed for the lung inhale insert. These differences may be related to the low density of the lung inhale tissue (0.204 g/cm^3^) [18], which leads to a low interaction probability of the CT x-ray with traversed tissue.

Moreover, slight variations in *μ*_measured_ lead to large errors due to the small *μ*_theoretical_ value for lung inhale (0.027 cm^−1^). However, a relative error of 18% (Additional file 1: Figure S2A) in the measured attenuation coefficient is associated with a relative error of only 3.5% (Fig. 7A) in the ACF-based activity calculation. One limitation of our experimental design is that lung tissue is represented only by a small insert, whereas the lungs cover a large region of the thorax in patients. However, ^177^Lu quantification in the lungs may be relevant only in patients presenting diffused lung uptake, which clinically can be a contraindication for the administration of a radionuclide therapy [27]. In addition, the observed overestimation of *μ*_measured_ for lung exhale and bone ($\sim$ 5%) does not introduce substantial errors ($\sim$ -2%) in the ACF-based ^177^Lu activity quantification.

Beyond an accurate activity quantification, quantitative ^177^Lu SPECT/CT should ideally provide enough anatomical information to enable CT-based organ and tumor segmentation in internal dosimetry procedures. Therefore, balancing image noise and CT absorbed dose is essential. In this study, a fast increase of CTDI_vol_ with a slight decrease in the noise COV was observed between the 80 kVp / 20 mAs and the 130 kVp / 35 mAs images. Moreover, the combinations with 110 kVp and 130 kVp, resulted in very similar noise levels (COV $\sim$ 3 × 10^–3^), indicating that they play a minor role in the selection of the kVp and QRM combination. The minimal discrepancies observed in CoV may be attributed to the technique used for generating the *μ*-map and the methodology employed for determining the CoV. The *μ*-map in the image space is generated in two main steps: First, the CT image is resampled to match the SPECT voxel size; second, a bilinear model is applied to establish a relationship between Hounsfield units (HUs) and attenuation coefficients [1, 3]. These steps result in *μ*-maps that have a smoother appearance compared to the underlying CT images.

In this study, the CoV was specifically calculated on the *μ*-map to directly assess its impact on the SPECT image (attenuation correction). Assessing the CoV using the CT images may not adequately account for the effects of the steps involved in generating the *μ*-map. Additionally, CoV was measured in the homogeneous region (plastic water) of the electron density phantom, where the attenuation coefficients are expected to remain in a very similar range. In addition, the positioning of the VOIs used for the quantification of the CoV was based on the noise assessment method recommended in the ICRU report 87 [2].

A comparison between the maximum CTDI_vol_ observed on this study (3.6 mGy, 130 kVp / 35 mAs) and the CTDI_vol_ values reported by Verfaillie *et al.* [7] for CT attenuation correction and anatomical localization (between 3.1 mGy for thyroid scans with ^99m^Tc and 4.9 mGy for parathyroid scans with ^99m^Tc) shows that all kVp and QRM combinations used in this study are in the low range of CTDI_vol_. Despite the generally low dose regime, the combination of 110 kVp / 20 mAs, featuring the best trade-off between noise (COV = 3.1 × 10^–3^) and CTDI_vol_ (1.5 mGy) (Fig. 7), offers the possibility of further dose reduction compared to the combination of 130 kVp / 35 mAs with the highest quality at maximum CTDI_vol_ (COV = 2.9 × 10^–3^, CTDI_vol_ = 3.6 mGy). However, the combination 130 kVp / 20 mAs (COV = 3.1 × 10^–3^, CTDI_vol_ = 2.0 mGy) could be also considered in large-sized patients or in studies in which the patient arms are in the field-of-view.

Three different reconstruction algorithms (xSPECT Quant, Flash3D, and STIR) were compared in the last section of this study. The generally poor spatial resolution of SPECT imaging can lead to incorrect attenuation correction especially for voxels located near the interface of two or more materials with different attenuation coefficients. This scenario was reproduced in this study by exploring the quantification at the interfaces of water/polystyrene (W/PS), water/polytetrafluoroethylene (W/PTFE), water/polyamide (W/PA), and water/polypropylene (W/PP). The largest differences in the activity quantification were expected for the first two interfaces because of the large change in density (W/PS, 1 g/cm^3^ to 0.023 g/cm^3^ [ratio: 43.5] and W/PTFE, 1 g/cm^3^ to 2.18 g/cm^3^ [ratio: 0.5]). These assumptions were confirmed experimentally, where the activity quantification generally showed the largest error at the W/PTFE interface for both syringe geometries.

Another parameter that affects the activity quantification is the voxel size. The three reconstruction algorithms can be divided into two groups based on the reconstructed voxel size: 1) xSPECT Quant (1.953 mm) and 2) Flash3D and STIR (both 4.8 mm). Therefore, the largest differences in the activity quantification were expected in the images reconstructed with a voxel size of 4.8 mm. Furthermore, this difference should be more relevant for the 1-mL syringe (0.7 cm diameter) than for the 10-mL syringe (1.6 cm diameter) as, for a voxel size of 4.8 mm, the 1-mL syringe consists of only two axial voxels, whereas the 10-mL source covers at least four axial voxels. This assumption may explain why xSPECT Quant provides a better activity quantification than Flash3D and STIR (Fig. 9 and Additional file 1: Figure S4). In case of Flash3D, at the W/PTFE interface, the combinations of 80 kVp / 35 mAs and 130 kVp / 35 mAs showed relative errors of 20.5% and 25.6%, respectively, while the others remained close to 50%. This difference may be related to a combination of the two discussed parameters: (1) Assigning an incorrect attenuation correction value to voxels near the interface and (2) the voxel size, which increases the possibility of voxels being averaged near the interface. Clinically, significant amounts of activity in small cavities (0.7 mm diameter) surrounded by high-density material (e.g., bone cavities) can occur in the ribs (spongiosa tissue), where the patient's breathing may introduce movement errors, or in small metastases/tumors close to a cortical bone structure. Further studies are planned in our institution to reproduce these conditions.

The last parameter to be considered for activity quantification is the partial volume effect (PVE) of the SPECT image. An increase in source size is associated with a reduction in the PVE of the SPECT image from 1-mL syringe sources to 10-mL syringe sources. In addition, the increase in source size increases the number of voxels with a correctly assigned attenuation correction value. The overall effect is a better activity quantification with the 10-mL syringes than with the 1-mL syringes. This is reflected in the relative error (Fig. 9 and Additional file 1: Figure S4), where each reconstruction algorithm is compared individually (1 mL syringes vs. 10 mL syringes).

The 4% improvement in ^177^Lu SPECT-based activity quantification for xSPECT Quant within the studied combinations of kVp and QRM for both source geometries may be related to the reduction in image noise of the µ-maps. This result may be relevant for bone marrow dosimetry (activity quantification in spongiosa tissue). However, this improvement may only play a minor role when considering other sources of error that occur in internal dosimetry calculations (e.g., regarding segmentation of volumes of interest, time-activity curve fitting, or S-value scaling). Therefore, an improvement of this magnitude should only be of minor importance when choosing between high and low kVp.

xSPECT Quant reconstructed images show the best activity quantification values across all attenuation materials and source geometries. Similarly, previous studies have shown that OSCGM reconstruction algorithms such as xSPECT Quant provide better spatial recovery of the activity than OSEM reconstruction algorithms [24]. This in turn leads to better image resolution, which together with a smaller voxel size (1.953 mm) than both OSEM-based algorithms, resulted in better material registration at the voxel level, overall better activity quantification, and smaller partial volume errors/effect than in images reconstructed with Flash3D or STIR.

## Conclusion

This study evaluated the effect of kVp and QRM on quantitative ^177^Lu SPECT/CT imaging. A methodology for the analysis of both parameters is described that can be implemented for other SPECT/CT system or for other radionuclides. Our study shows that kVp and QRM in the typical range of low-dose CT have a minimum effect on activity quantification based on quantitative ^177^Lu SPECT/CT. Additionally, it was demonstrated that by choosing an appropriate combination of kVp and QRM, the CT fraction of the absorbed dose in quantitative SPECT/CT acquisitions can be reduced without significantly increasing image noise. Finally, reconstructions with a better image resolution were found to be less likely to assign activity to the wrong material on a voxel level—resulting from better image resolution and, thus, smaller partial volume errors.

### Supplementary Information

**Additional file 1:** Supplementary data.

**Additional file 2:** ACF calculation for a single voxel.

**Additional file 3:** ACF calculation for 80 kVp / 20 mAs attenuation coefficient map.

## Data Availability

Images of the digital phantom and Python code can be requested from the corresponding author.

## Abbreviations

kVp: Kilovoltage peak
mAs: Product of tube current and tube-on time per rotation
QRM: Quality reference mAs
CTDI_vol_: CT dose index volume
VOI: Volume of interest
COV: Coefficient of variation
CT: Computer Tomography
PET: Positron Emission Tomography
SPECT: Single Photon Emission Tomography
DRLs: Diagnostic reference levels
AC: Attenuation correction
CT-AC: CT for attenuation correction
ACF: Attenuation correction factor
PS: Polystyrene
PTFE: Polytetrafluoroethylene
PA: Polyamide
PP: Polypropylene
