# Supplementary material for: Effect of kilovoltage and quality reference mAs on CT-based attenuation correction in 177Lu SPECT/CT imaging: a phantom study
Source: EJNMMI Phys. 2024 Feb 26;11:21. doi: 10.1186/s40658-024-00622-6 (PMC11266317; doi:10.1186/s40658-024-00622-6)
Supplement: Supplementary file 1 — Additional file 1: Supplementary data. [file 40658_2024_622_MOESM1_ESM.docx]

**Effect of Kilovoltage and Quality Reference mAs on CT-Based Attenuation Correction in 177Lu SPECT/CT Imaging: a phantom**

**Author Names and Degrees:**

Dr. Maikol Salas-Ramirez^1^*,

MSc. Julian Leube^1^,

Prof. Dr. Michael Lassmann^1^,

Dr. Johannes Tran-Gia^1^

**Affiliations:**

^1^Department of Nuclear Medicine, University Hospital Würzburg, Würzburg, Germany

DERIVATION OF EQUATION 1

The change in ^177^Lu activity quantification ($\Delta A[\%]$) is defined as:

$\text{∆A}\left( {}^{\text{177}}\text{Lu} \right)\text{[\%]=}\left( \frac{\text{A}_{\text{measured}}}{\text{A}_{\text{theoretical}}}\text{-1} \right)\text{∙100\%}$ [S1]

Here, $\text{A}_{\text{measured}}$ is the activity measured using μ_measured_-map. $\text{A}_{\text{theoretical}}$ is the activity measured using μ_theoretical_-map_._

Next, the non-attenuated activity ($\text{A}_{\text{0}}$, true activity) is introduced:

$\text{∆A}\left( {}^{\text{177}}\text{Lu} \right)\text{[\%]=}\left( \frac{\frac{\text{A}_{\text{measeured}}}{\text{A}_{\text{0}}}}{\frac{\text{A}_{\text{theoretical}}}{\text{A}_{\text{0}}}}\text{-1} \right)\text{∙100\%}$ [S2]

By definition $\text{ACF}_{\text{measured}}\text{=}\frac{\text{A}_{\text{0}}}{\text{A}_{\text{measured}}}$ and $\text{ACF}_{\text{theoretical}}\text{=}\frac{\text{A}_{\text{0}}}{\text{A}_{\text{theoretical}}}$. Therefore, the equation 2 takes the form:

$\text{∆A}\left( {}^{\text{177}}\text{Lu} \right)\text{[\%]=}\left( \frac{\frac{\text{1}}{\text{ACF}_{\text{measured}}}}{\frac{\text{1}}{\text{ACF}_{\text{theoretical}}}}\text{-1} \right)\text{∙100\%}$ [S3]

Lastly, the equation 1 in the manuscript is obtained:

$\text{∆A}\left( {}^{\text{177}}\text{Lu} \right)\text{[\%]=}\left( \frac{\text{ACF}_{\text{theoretical}}}{\text{ACF}_{\text{measured}}}\text{-1} \right)\text{∙100\%}$ [S4]

SUPPLEMENTAL TABLES

Supplemental table 1. Nominal activities inside of the syringes

| Source geometry  (Syringe volume, mL) | Attenuation  material | Nominal activity (kBq) |
| --- | --- | --- |
| 1 | PS | 146 |
|  | PTFE | 162 |
|  | PP | 168 |
|  | PA | 155 |
| 10 | PS | 1587 |
|  | PTFE | 1524 |
|  | PP | 1443 |
|  | PA | 1505 |

SUPPLEMENTAL FIGURES


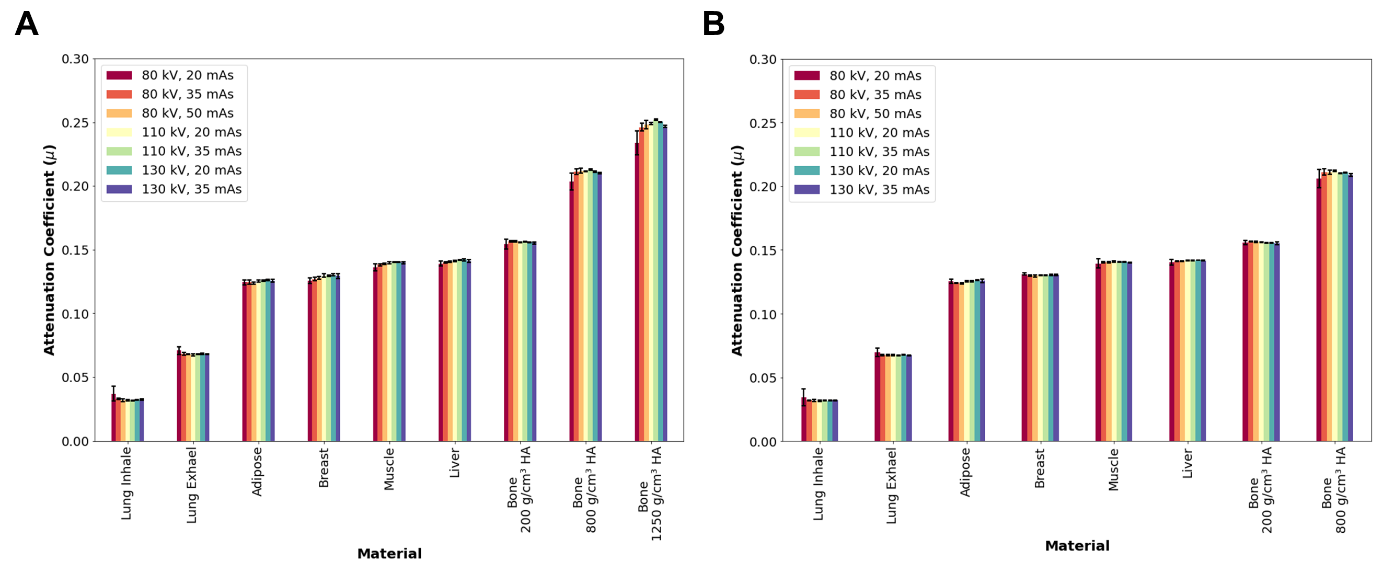


Supplemental Figure 1. Measured attenuation coefficient (µ_measured_) of the ^177^Lu µ-map for the seven studied combinations of kVp and QRM: A) Inner inserts. B) Outer inserts.


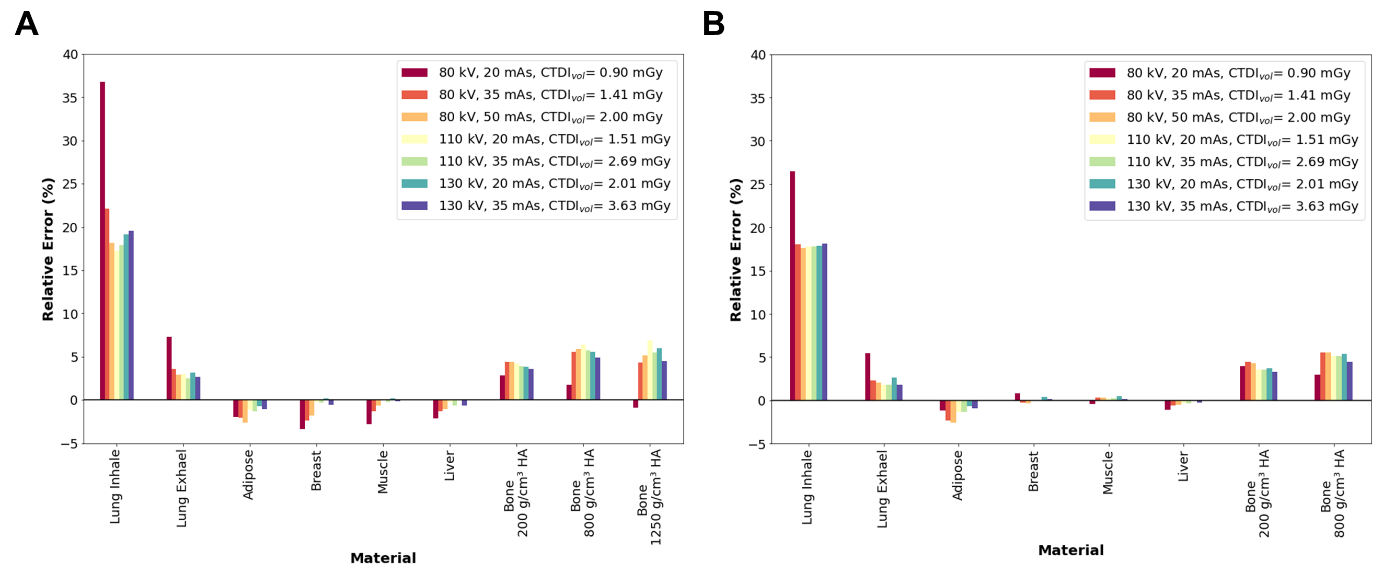


Supplemental Figure 2. Relative errors between measured (µ_measured_) and theoretical (µ_theoretical_) attenuation coefficients of ^177^Lu µ-map for the seven studied combinations of kVp and QRM: A) Inner inserts. B) Outer inserts.


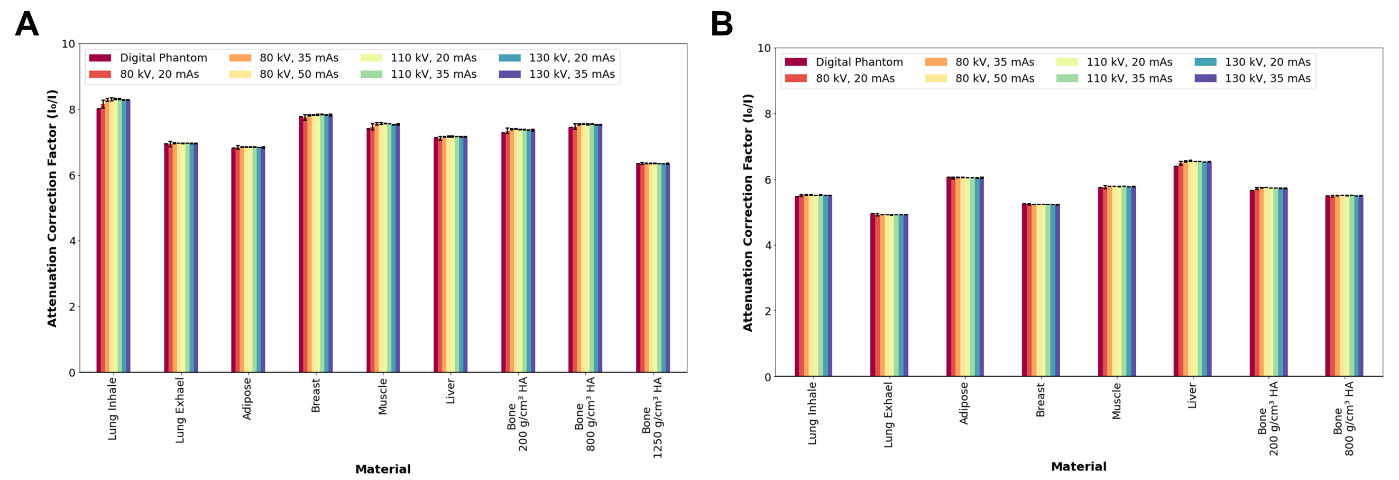


Supplemental Figure 3. Measured and theoretical ACF values: A) Intern inserts. B) Extern inserts. $I_{0}$ is the image with attenuation correction and $I$ is the image without attenuation correction. Theoretical ACF values are represented by the digital phantom.
