# Supplementary material for: Effect of kilovoltage and quality reference mAs on CT-based attenuation correction in 177Lu SPECT/CT imaging: a phantom study
Source: EJNMMI Phys. 2024 Feb 26;11:21. doi: 10.1186/s40658-024-00622-6 (PMC11266317; doi:10.1186/s40658-024-00622-6)
Supplement: Supplementary file 2 — Additional file 2: ACF calculation for a single voxel. [file 40658_2024_622_MOESM2_ESM.pdf]

This code shows how the ACF is calculated for a single voxel of the digital phantom

In [1]:
```
# General used library list
import SimpleITK as sitk
import numpy as np
from __future__ import division
from os import mkdir
from os.path import join, isdir
from imageio import imread, imwrite
import matplotlib.pyplot as plt
import astra
import pydicom as dcm
import tomopy
import ipywidgets as ipw
import numpy.ma as ma
from skimage.morphology import disk
from skimage import morphology
import glob
import pydicom
from scipy.ndimage import zoom
import nrrd
import scipy
from scipy import ndimage
from PIL import Image
from scipy.interpolate import interp1d
```

In [2]:
```
# This funtion allows to plot the phantom images
def plot_image(tran, coro, sagi):
    mini = image.min()
    maxi = image.max()
    cmap = 'gray'

    plt.figure(figsize=(16, 4))
    a1=plt.subplot(1, 3, 1)
    plt.imshow(np.rot90(np.rot90(image[tran, :, :])), origin='lower', cmap=cmap, vmin=mini, vmax=maxi)
    a1.set_aspect(cor_aspect_mu)
    plt.xticks(fontsize=14)
    plt.yticks(fontsize=14)
    #plt.colorbar(pad=0.02)
    cb = plt.colorbar(pad=0.02)
    font_size = 14 # Adjust as appropriate.
    cb.ax.tick_params(labelsize=font_size)

    a2=plt.subplot(1, 3, 2)
    plt.imshow(np.rot90(np.rot90(image[:, coro, :])), origin='lower', cmap=cmap, vmin=mini, vmax=maxi)
    a2.set_aspect(sag_aspect_mu)
    cb = plt.colorbar(pad=0.02)
    font_size = 14 # Adjust as appropriate.
    cb.ax.tick_params(labelsize=font_size)
    plt.xticks(fontsize=14)
    plt.yticks(fontsize=14)


    a3=plt.subplot(1, 3, 3)
    plt.imshow(np.rot90(np.rot90(image[:, :, sagi])), origin='lower', cmap=cmap, vmin=mini, vmax=maxi)
    a3.set_aspect(ax_aspect_mu)
    cb = plt.colorbar(pad=0.02)
    font_size = 14 # Adjust as appropriate.
    cb.ax.tick_params(labelsize=font_size)
    plt.xticks(fontsize=14)
    plt.yticks(fontsize=14)
    plt.show()

    return;
```

Please copy the funtions: euclideanDistance, getLinecut, getRowCol, binarySearch, exclusionCondition, getEdgePointsAcrossCenter, and radialAverage from: https://github.com/xuejianma/fastLinecut_radialLinecut/blob/main/.ipynb_checkpoints/fastLinecut_radialLinecut-checkpoint.ipynb (https://github.com

/xuejianma/fastLinecut_radialLinecut/blob/main/.ipynb_checkpoints/fastLinecut_radialLinecut-checkpoint.ipynb)

In [3]:
```
def euclideanDistance(coord1,coord2):

def getLinecut(image,X,Y,pt1,pt2):

def getRowCol(pt,X,Y):

def binarySearch(left,right,conditionFunction,threshold=1e-5):

def exclusionCondition(pt,X,Y):

def getEdgePointsAcrossCenter(image,X,Y,center,angleDegree):

def radialAverage(graph,center,X,Y,angleSteps,angleOffsetDegree = 0):
```

Here the attenuation coefficient image from the digital phantom image is opened

In [4]:
```
path_FILE = '/home/nuksimu/Documents/CT_110/Noise_Analysis/Segment_Digital_Phantom'
```

In [5]:
```
data_digiphantom,header_digiphantom = nrrd.read(path_FILE+'/new_segmentation_3.nrrd')
```

In [6]:
```
# The image is reoriented
data_digiphantom_t = np.flip(np.rot90(np.rot90(np.rot90(data_digiphantom), k=1, axes=(2, 1)), k=1, axes=(0, 1)))
data_digiphantom_t.shape
```

Out[6]:
```
(53, 128, 128)
```

In [7]:
```
# Calculate the image display factors (voxel scaling factor) needed in the funtion "plot_image"
ax_aspect_mu = header_digiphantom['space directions'].flat[4]/header_digiphantom['space directions'].flat[0]
cor_aspect_mu = header_digiphantom['space directions'].flat[4]/header_digiphantom['space directions'].flat[8]
sag_aspect_mu = header_digiphantom['space directions'].flat[4]/header_digiphantom['space directions'].flat[8]
print(ax_aspect_mu,cor_aspect_mu,sag_aspect_mu)
```

```
1.0 1.0 1.0
```

In [8]:
```
# Plot phantom image
image = data_digiphantom_t
ipw.interact(plot_image, tran=(0, image.shape[0]-1), coro=(0, image.shape[1]-1), sagi=(0, image.shape[2]-1));
```

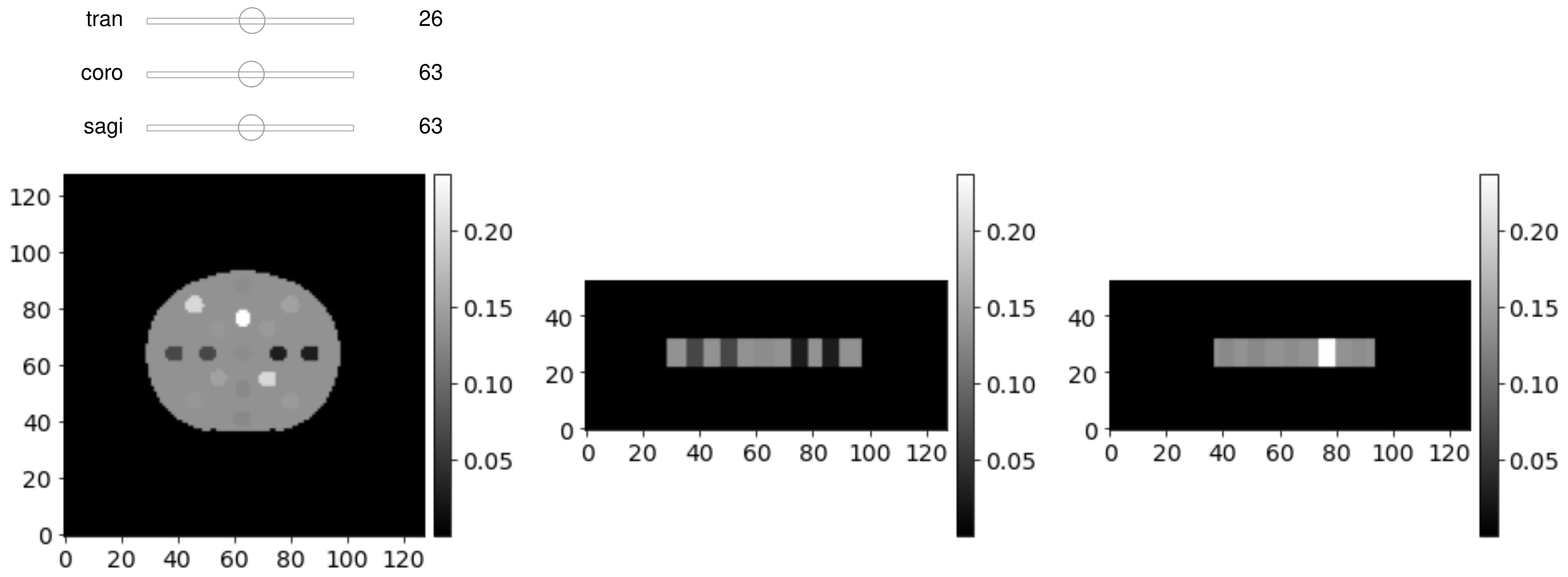

Here is created the phantom mask based on a threshold of 0.02

In [9]:
```
#This mask define the limits in the image where the Chang method is applied
t = 0.02
mask = data_digiphantom_t > t
```

In [10]:
```
# Plot mask image
image = mask
ipw.interact(plot_image, tran=(0, image.shape[0]-1), coro=(0, image.shape[1]-1), sagi=(0, image.shape[2]-1));
```

tran 26

coro 63

sagi 63

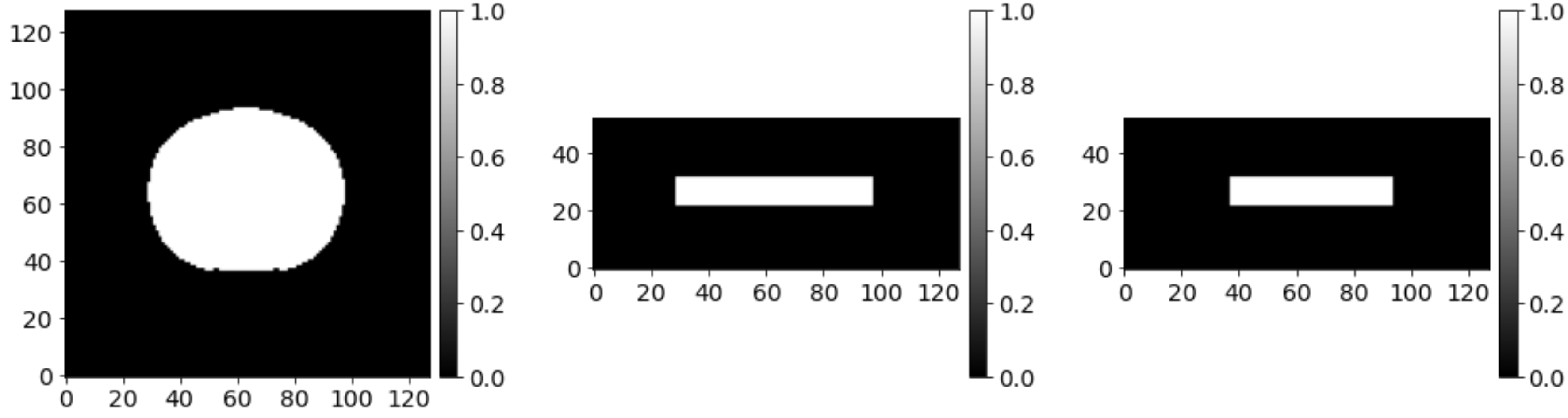

Here the ACF for one single voxel positioned in (64,64) and slice 25 is calculated

In [11]:
```
n = 25
image = data_digiphantom_t[n,:,:]*mask[n,:,:]
binary_mask = mask[n,:,:]
image_copy = image
sx, sy = image_copy.shape
print(sx,sy)
X, Y = np.ogrid[0:sx, 0:sy]
numLines = 64
ACF = np.zeros(image_copy.shape)
center = (64,64)
combinedDistArray,combinedLinecut,edgePtsDB = radialAverage(image_copy,
                                                            center,X,Y,
                                                            numLines,
                                                            angleOffsetDegree=0)

ACF = 1/(np.exp(-0.5*np.sum(combinedLinecut*(combinedDistArray[1]-combinedDistArray[0])*0.4795)))
```

```
128 128
```

Here the radial profile is plotted

In [12]:
```
plt.figure(figsize=(7,7))
plt.xlabel('Radial distance (cm)', fontsize=20,fontweight='bold')
plt.ylabel('Attenuation Coefficient ($\mu$) (1/cm)', fontsize=20,fontweight='bold')
plt.xticks(fontsize=18)
plt.yticks(fontsize=18)
plt.plot(combinedDistArray,combinedLinecut)
```

Out[12]:
```
[<matplotlib.lines.Line2D at 0x7fa06159bc70>]
```

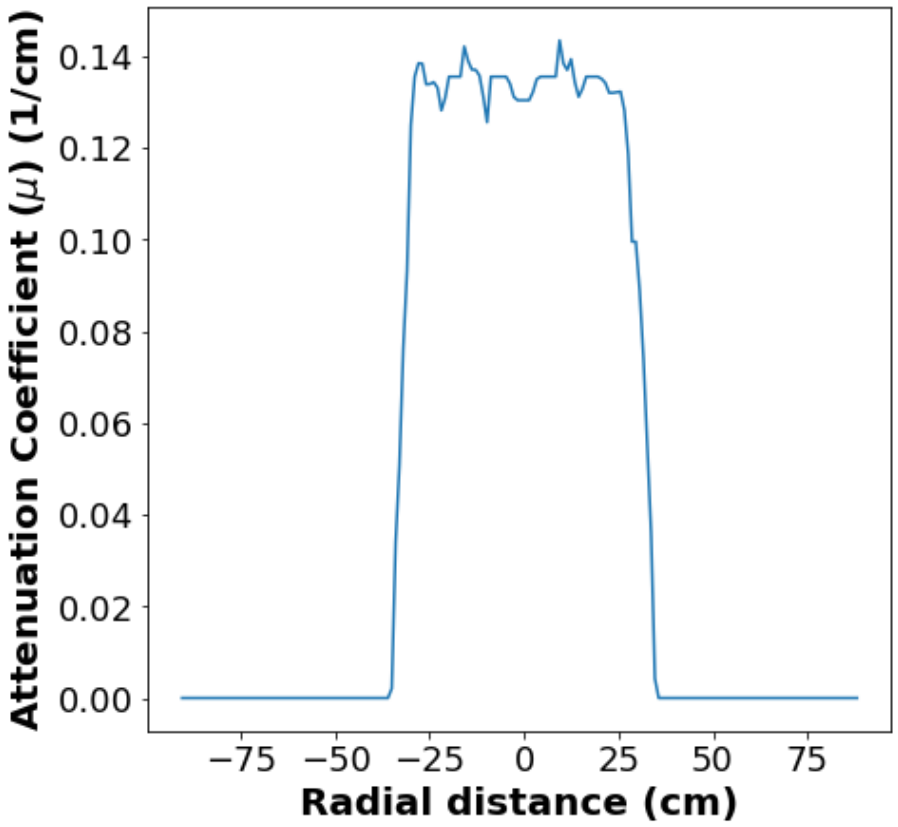

Here are ploted all the sampling profiles used to generate the previous profile

In [13]:
```
plt.figure(figsize=(7,7))
plt.gca().set_aspect(1)
plt.xticks(fontsize=18)
plt.yticks(fontsize=18)
for ind in range(len(edgePtsDB)):
    edgeNeg,edgePos = edgePtsDB[ind]
    plt.plot([edgeNeg[0],edgePos[0]],[edgeNeg[1],edgePos[1]])
```

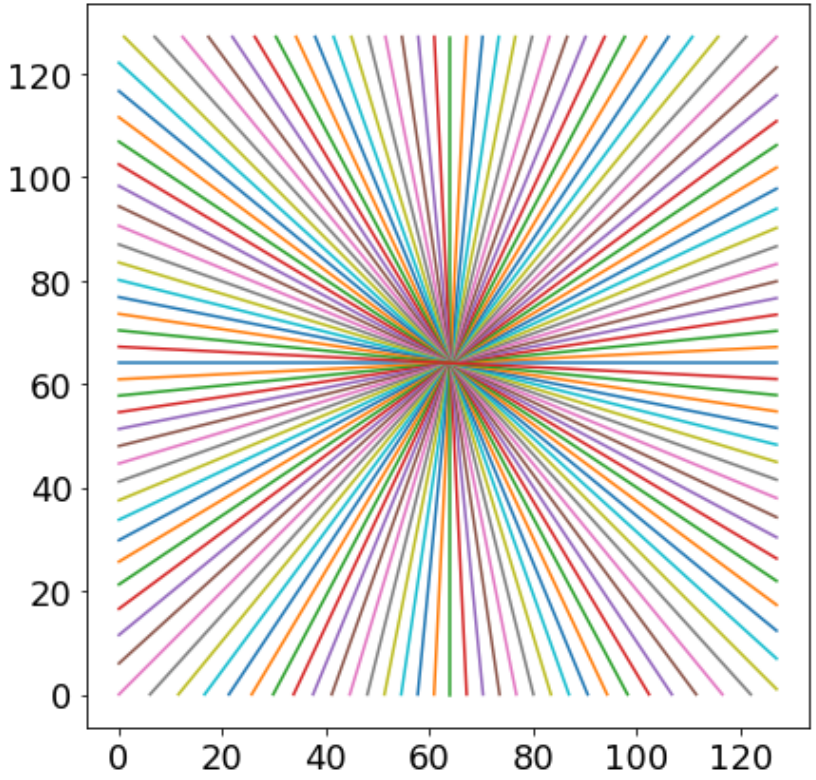

In [ ]:
