# Supplementary material for: Effect of kilovoltage and quality reference mAs on CT-based attenuation correction in 177Lu SPECT/CT imaging: a phantom study
Source: EJNMMI Phys. 2024 Feb 26;11:21. doi: 10.1186/s40658-024-00622-6 (PMC11266317; doi:10.1186/s40658-024-00622-6)
Supplement: Supplementary file 3 — Additional file 3: ACF calculation for 80 kVp / 20 mAs attenuation coefficient map. [file 40658_2024_622_MOESM3_ESM.pdf]

This code shows how the ACF for the 80 kVp / 20 mAs image was generated

In [ ]:
```
# General used library list
import SimpleITK as sitk
import numpy as np
from __future__ import division
from os import mkdir
from os.path import join, isdir
from imageio import imread, imwrite
import matplotlib.pyplot as plt
import astra
import pydicom as dcm
import tomopy
import ipywidgets as ipw
import numpy.ma as ma
from skimage.morphology import disk
from skimage import morphology
import glob
import pydicom
from scipy.ndimage import zoom
import nrrd
import scipy
from scipy import ndimage
from PIL import Image
from scipy.interpolate import interp1d
```

Please copy the funtions: euclideanDistance, getLinecut, getRowCol, binarySearch, exclusionCondition, getEdgePointsAcrossCenter, and radialAverage from: https://github.com/xuejianma/fastLinecut_radialLinecut/blob/main/.ipynb_checkpoints/fastLinecut_radialLinecut-checkpoint.ipynb (https://github.com/xuejianma/fastLinecut_radialLinecut/blob/main/.ipynb_checkpoints/fastLinecut_radialLinecut-checkpoint.ipynb)

In [ ]:
```
def euclideanDistance(coord1,coord2):

def getLinecut(image,X,Y,pt1,pt2):

def getRowCol(pt,X,Y):

def binarySearch(left,right,conditionFunction,threshold=1e-5):

def exclusionCondition(pt,X,Y):

def getEdgePointsAcrossCenter(image,X,Y,center,angleDegree):

def radialAverage(graph,center,X,Y,angleSteps,angleOffsetDegree = 0):
```

Open attenuation coefficients images (u-maps)

In [ ]:
```
i=0
for i in range(10):
    locals()["File_80_20_"+str(i+1)] = dcm.read_file("PATH_to/80_20/"+str(i+1)+".dcm")
```

Apply the scaling factor (10000) specified in the dicom header of the image.

In [ ]:
```
i=0
for i in range(10):
    locals()["arr_image_80_20_"+str(i+1)] = locals()["File_80_20_"+str(i+1)].pixel_array/100000
```

Define the array data type

In [ ]:
```
i=0
for i in range(10):
    locals()["arr_image_80_20_"+str(i+1)] = locals()["arr_image_80_20_"+str(i+1)].astype(float)
```

Open the digital phanton to create a mask

In [ ]:
```
path_FILE = 'PATH_to/Segment_Digital_Phantom'
data_digiphantom,header_digiphantom = nrrd.read(path_FILE+'/Digital_Phantom.nrrd')
# The image is reoriented
data_digiphantom_t = np.flip(np.rot90(np.rot90(np.rot90(data_digiphantom), k=1, axes=(2, 1)), k=1, axes=(0, 1)))
```

Create a mask from digital based on the threshold that select all material diferent to air

In [ ]:
```
#This mask define the limits in the image where the Chang method is applied
t = 0.02
mask = data_digiphantom_t > t
```

Here the ACF for the phantom voxels are calculated for the 10 slides that form the insert of the Electron Density Phantom

In [ ]:
```
i=0
for i in range(10):
    arraysDict = {}
    for n in range(20,31):
        image = locals()["arr_image_80_20_"+str(i+1)][n,:,:]*mask[n,:,:]
        binary_mask = mask[n,:,:]
        image_copy = image
        sx, sy = image_copy.shape
        X, Y = np.ogrid[0:sx, 0:sy]
        numLines = 64
        ACF = np.zeros(image_copy.shape)
        for l in range(image_copy.shape[0]):
            for m in range(image_copy.shape[1]):
                if binary_mask[l,m]>0:
                    center = (l,m)
                    combinedDistArray,combinedLinecut,edgePtsDB = radialAverage(image_copy,center,X,Y,
                                                                                numLines,
                                                                                angleOffsetDegree=0)
                    ACF[l,m] = 1/(np.exp(-0.5*np.sum(combinedLinecut*
                                                     (combinedDistArray[1]-combinedDistArray[0])*0.4795)))
                                                        #0.4795 is the voxel size
        arraysDict['ACF_{0}'.format(n)] = ACF

    locals()["ACF_80_20_"+str(i+1)] = np.rot90(np.rot90(np.dstack(arraysDict.values()), k=1, axes=(1, 2)))
```

Save ACF images

In [ ]:
```
i=0
for i in range(10):
    output = locals()["ACF_80_20_"+str(i+1)].astype(np.float32)
    output = sitk.GetImageFromArray(output)
    output.SetSpacing([pixel_mu[1],pixel_mu[1],pixel_mu[1]])
    sitk.WriteImage(output, "PATH_to/I_80_20/"+str(i+1)+".mhd")
```

In [ ]:
